# Want to quickly adapt to distorted speech and become a better listener? Read lips, not text

**DOI:** 10.1371/journal.pone.0278986

**Published:** 2022-12-29

**Authors:** Faezeh Pourhashemi, Martijn Baart, Thijs van Laarhoven, Jean Vroomen

**Affiliations:** 1 Dept. of Cognitive Neuropsychology, Tilburg University, Tilburg, The Netherlands; 2 BCBL, Basque Center on Cognition, Brain, and Language, Donostia, Spain; CNRS - Université d’Aix-Marseille, FRANCE

## Abstract

When listening to distorted speech, does one become a better listener by looking at the face of the speaker or by reading subtitles that are presented along with the speech signal? We examined this question in two experiments in which we presented participants with spectrally distorted speech (4-channel noise-vocoded speech). During short training sessions, listeners received auditorily distorted words or pseudowords that were partially disambiguated by concurrently presented lipread information or text. After each training session, listeners were tested with new degraded auditory words. Learning effects (based on proportions of correctly identified words) were stronger if listeners had trained with words rather than with pseudowords (a lexical boost), and adding lipread information during training was more effective than adding text (a lipread boost). Moreover, the advantage of lipread speech over text training was also found when participants were tested more than a month later. The current results thus suggest that lipread speech may have surprisingly long-lasting effects on adaptation to distorted speech.

## Introduction

Human speech is sometimes difficult to understand due to background noise, an unfamiliar accent of the speaker, or poor quality of the speech signal itself. However, listeners quickly adapt to this situation because there is often other information available that informs the listeners what the intended spoken message should be. This ‘other’ information might be lipread speech if the speaker can be seen, or it might consist of subtitles that can be read while speech is heard when watching a movie [e.g., 1–3].

Knowledge about the possible words in a language is also important for disambiguating the intended message. For example, the Ganong effect shows that an ambiguous auditory phoneme is perceived as /g/ when followed by ‘ift’, but perceived as /k/ when followed by ‘iss’ because only ‘gift’ and ‘kiss’ are legal words in English [4]. Here, we hypothesized that these extra information sources–lipread speech, written text, and lexical knowledge–all provide a basis for determining the discrepancy between the intended and actual speech signal that guides adaptive changes in speech perception. However, until now, their effectiveness has never been directly compared in a systematic way. Here, we therefore examined whether lipread speech, written text, and lexical information differ in their efficacy to drive learning of distorted speech. Remarkably, the least constraining and, arguably, most difficult information source to decode–lipread speech–proved to be the most effective teacher.

Learning effects in distorted speech have been demonstrated many times, and can even occur without other information sources that disambiguate the distorted signal [e.g., 5, 6]. Also, mere exposure to clear but accented speech results in improvements in performance in the absence of other information about the correct interpretation [e.g. 7–9]. Simply listening to time-compressed speech [8–10] can also lead to intelligibility improvements. However, there is no doubt that speech perception also makes use of other information sources to adapt or ‘recalibrate’ auditory perception [11–16]. One example comes from the literature on ‘*phonetic recalibration*’ where ambiguous phonetic segments are adjusted so that they fit in the context. For lipread speech, this was first demonstrated by Bertelson et al. [12] who exposed listeners to the view of a speaker who pronounced either /aba/ or /ada/ while an ambiguous speech sound halfway between /aba/ and /ada/ was heard. In auditory-only posttests, identification of the ambiguous sound was shifted towards the previously seen lipread information, so the same test sound was perceived more likely as /aba/ when the previous exposure contained lipread /aba/, and more likely as /ada/ when the previous exposure contained lipread /ada/. The rationale behind this assimilative effect is that the perceptual system during audiovisual exposure minimizes the discrepancy between the heard and seen information by shifting the auditory phonetic boundary towards the lipread stimulus. This then leads to assimilative auditory aftereffects in posttests.

Similar, but generally smaller assimilative aftereffects have been reported when instead of lipread speech, written text [17] or lexical information [16, 18–20] is used as the teacher-signal that disambiguates the ambiguous phoneme. The reason why lipread speech is more potent than text or lexical information remains, for the time being, rather elusive. On the one hand, it seems that lipread speech is biologically closely tied to auditory speech production. Reading, on the other hand, has emerged only late in evolution and our ability to read is typically built on our ability to process auditory speech. However, this does not imply that lipread speech should, by default, be more helpful than text because the mapping between lipread visemes and auditory phonemes is not as clear as mapping between phonemes and text. For example, the visible articulatory movements that correspond to /b/ (i.e., a bilabial closure) can easily be mistaken for /p/ or /m/, and even professional lipreaders struggle with decrypting silent lipread videos [21]. In contrast, for typical readers of an alphabetical script, the mapping between orthography and phonology is constrained and leaves little room for visual-auditory ambiguities (at least not in a language with a relatively transparent orthography like Dutch). Nevertheless, only dynamic lipread information is tightly correlated with the unfolding auditory speech stream [e.g., 22, 23], which may explain why silent lip-read information can activate auditory cortex [24] and can drive so-called ‘cortical entrainment’ in which oscillatory cortical activity in auditory areas synchronizes with the (silent) moving lips [25, 26].

Lexical context (rather than orthographic context), may, arguably, also be more constraining than lipread speech: in the previously mentioned Ganong-effect, only the words ‘gift’ or ‘kiss’ provided a fully constraining context when /?ift/ or /?iss/ was presented to the listeners. Despite these differences in biology, acquisition, and information content, though, it has been repeatedly demonstrated that all three information sources induce phonetic recalibration, although lipread information seems more potent in inducing this recalibration effect on phoneme identification.

Learning effects of lipread speech, written text, and lexical context have also been found in studies on *spoken word recognition* where instead of ambiguous phonemes, longer segments like words or sentences are used. One striking example from the literature on spoken word recognition comes from noise-vocoded speech in which spectral (and some temporal) details from the speech signal are removed [27]. When heard for the first time, noise-vocoded speech sounds rather unintelligible, but intelligibility drastically improves if the content of the sentence is revealed by a clear (undistorted) speech example. The second presentation of the vocoded sentence then seems much more intelligible than on initial hearing. Davis et al. [5] examined whether learning to identify noise-vocoded speech differed if the identity of the distorted items was revealed via a clear speech example or concurrently presented written text. Their results showed that both information sources were equally effective. Presumably, clear speech prior to, or written presentation concurrent with distorted speech presentation drove learning by providing the correct target representation. Furthermore, they reported that learning effects were absent when the distorted target sentence contained pseudowords instead of real words, thus suggesting that lexical context matters. However, the training sentences themselves may contain sentence-level information that can boost identification of test items. This potential issue was acknowledged in a follow-up study by the same group [28], and was circumvented by using single items rather than full sentences. However, in that study, text was no longer included as a training context (learning was assessed via clear speech feedback only), and the only other study we are aware off that investigated text-based learning in NVS again used full sentences rather than single items [29]. Pilling et al. [29] did compare the boosting effect of written text versus lipread speech and showed that both visual contexts were equally effective in driving learning. Note that this result is somewhat different from the one obtained in phonetic recalibration with single items, where lipread speech is usually more potent than text, but again, in the work by Pilling et al. [29], the effect of sentence-level content during training (and its potential interaction with lipread information or text) could not be ruled out.

Here, we systematically examined, for the first time, whether lipread speech, printed text, and lexical information are able to boost auditory-only noise-vocoded speech identification when the training items consist of mono- or bi-syllabic noise-vocoded words or pseudowords. By using single items rather than sentences, we could assess the role of lexical information without sentence-level perceptual support (which can, for example, induce semantic carry-over effects between items), and rule out potential differences in parsing difficulty of connected speech that contains words versus pseudowords (or in the degree to which short-term memory is taxed).

In the current study, we used a training-test paradigm in which we measured recognition of auditory-only noise-vocoded words during a pretest followed by three short training blocksand test blocks. The training items and test items were all novel and were thus never repeated during the experiment. The training stimuli consisted of noise-vocoded words or pseudowords that were combined with either lipread speech, printed text, or a still frame of the speaker that served as a baseline condition. Training and test blocks always contained 15 items which allowed us to determine not only the overall effect of training context, but also how learning effects would build-up over time. In general, adaptation to distorted speech occurs rather rapidly [30], and our experimental design thus allowed us to compare adaptation when visual information is not informative (the still frame condition) with conditions where the visual context may boost learning via audiovisual integration (the text and lipread conditions).

We expected that training with words relative to pseudowords would boost auditory learning because lexical information provides top-down information that can drive learning (a lexical boost). As noted, both text and lipread information can drive perceptual learning (phonetic recalibration, see e.g., [12, 17]) so we also expected that lipread speech and text during training would boost learning relative to the static control condition. The relative contributions of lipread speech and text that is combined with single words or pseudowords during training had not been examined explicitly and thus was uncharted terrain. In Experiment 1, we assessed lexical, lipread and text based learning effects immediately after training, while in Experiment 2 we examined, in a subset of the participants (those who had trained with words), whether learning effects were stable and would last more than a month after the initial training-test procedure was completed.

## Experiment 1

### Materials and methods

#### Participants

One hundred and twenty-eight students from Tilburg University participated in this study in return for course credits. All participants were native speakers of Dutch who reported normal hearing and (corrected to) normal vision. Two participants in the word-training group were excluded from the analyses because more than 30% of the responses could not be analyzed (i.e., no response was provided, or responses were given in a different language than Dutch despite clear instructions). Also, one participant from the pseudoword- training group in the text condition was excluded because he/she had also participated in the word- training group. Including this participant in the data analyses did not change the patterns of (non) significance. Mean age in the final sample (92 females) was 19 years (SD = 1.70). The experiment was conducted in accordance with the Declaration of Helsinki. All participants provided written informed consent and the experiment was approved by the Tilburg University Ethics Review Board (project ID: EC-2016.48). The raw (concatenated) data is available for download from the DataverseNL platform (https://doi.org/10.34894/2D83AI).

#### Stimuli

**Fig 1:**
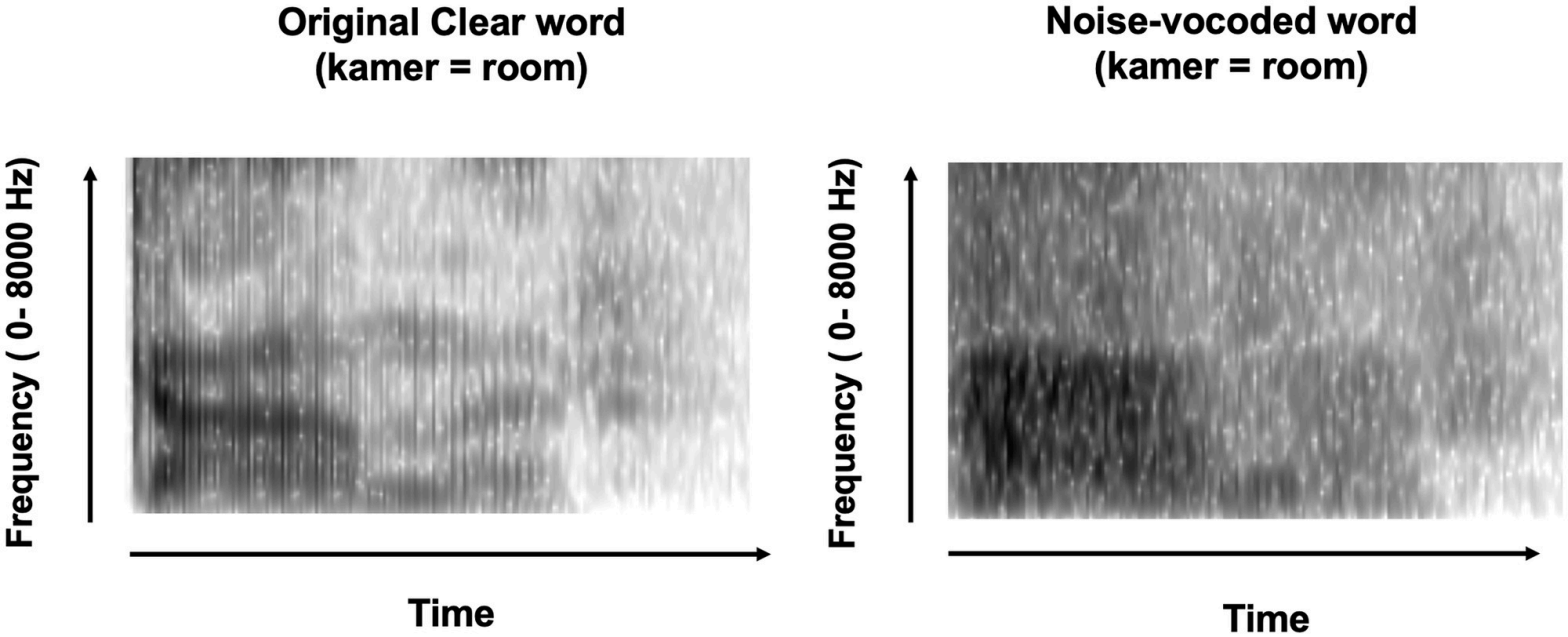
Example spectrograms. Original and noise-vocoded spectrogram for the item ‘kamer’.

For the auditory stimuli, a male native speaker of Dutch (MB, one of the authors) was recorded with a NIKON D7200 camera while pronouncing a set of 120 mono- and bi- syllabic Dutch words. This set (but different recordings) was originally used by van der Zande et.al. [31], and the same set of items was later used by van Laarhoven et al. [32]. The items had a mean word frequency of 153.75 per million, as determined with the SUBTLEX-NL database [33], see Supporting information for the full list (S1 Table). For training with pseudowords, a set of 120 pseudowords was recorded by the same speaker. The pseudowords were created by switching consonant positions within an item (e.g., “fout” [error] was changed into “touf”), or replacing a particular consonant with another one from the same place of articulation category (e.g., “kamer” [room] was changed into “pamer”). There were nine items for which these procedures could not yield pseudowords, and for these, consonants were swapped across items (e.g., “kip” [chicken] and “vijf” [five] were changed into “kif” and “vijp”) or a consonant was replaced with one from a different place of articulation category (“bom” [bomb] was changed into “nom”). Video recordings (25 f/s) were framed as headshots and included the entire face (from shoulders upwards, including the speaker’s hair) against a black background. The audio was recorded with an external microphone attached to the camera (RØDE VideoMicro). Individual clips of each item were extracted with Adobe Premiere Pro. The extracted audio was manipulated in the Praat software [34], using the Shannon-AM-noise script by Darwin [35]. Specifically, we created 4-channel noise-vocoded speech as follows: each auditory signal was decomposed into four non-overlapping frequency bands (50–800 Hz, 800–1500 Hz, 1500–2500 Hz, and 2500–4000 Hz). Next, for each band, the amplitude envelope was extracted and combined with a Hann band-pass filtered white noise signal (the smoothing value was defined as the highest frequency/10, so including filter skirts, the filtered bands now overlapped at the frequency band boundaries) and the bands were recombined into a 4-channel noise-vocoded speech stimulus [2], see Fig 1.

The rationale for using this type of NVS was that potential context-driven auditory learning effects are, generally, maximal when the auditory signal is poor, but still contains sufficient information to allow for context-driven perceptual restoration. Prior research has indicated that NVS word identification accuracy at least doubles when moving up from 2 to 4 channels, but then increases with about half that effect size when moving from 4 to 8 channels (where accuracy tapered-off and was comparable to 16 and 32 channel NVS, [36]). Moreover, two-year-olds start to show the first signs of word recognition with 4-channel NVS (when compared to 2-channel NVS, [37]), and Senan et al. [38] showed that dual task interference from 4-channel NVS was in between, but statistically comparable to 2-channel and 6-channel NVS (the primary task was a digit-recall task), whereas interference from 4 and 6-channel NVS was also statistically comparable to natural speech. We thus assumed that 4-channel NVS has a relatively poor intelligibility, but at the same time, might contain sufficient spectral detail to accommodate context-driven learning.

For the audiovisual training stimuli, the noise vocoded audio tracks were dubbed onto three different types of visual stimuli to create the training set: 1) the original lipread video of the speaker (size: 1920 px wide × 1080 px high, 2) printed text of the original word (Courier New font, bold typeface, size: 18 pts) and 3) a static image (referred to as still face) of the speaker (size: 1920 px wide x 1080 px high) that served as baseline. In the text condition, the orthographic item was presented 1800 ms before onset of the noise-vocoded audio and remained on the screen for the entire duration of the auditory stimulus. This was done to ensure that listeners had ample time to read the word/pseudoword, and activate the corresponding (phonological) and lexical code, before and during its auditory presentation. Because noise-compensation in audiovisual word processing may occur at a lexical/semantic level of processing [39], providing (more than) sufficient time to process the text before/during the AV presentation should provide participants with an optimal condition to learn from the text.

For both the word and psuedowowrd condition, all 120 items were randomly distributed across 15-item blocks. One of these was selected as the designated auditory-only familiarization block that was administered for all participants. The remaining 7 blocks of items were, for each participant, presented in random order, and randomly assigned to the AV training or auditory-only test procedures. Moreover, within each block, item order was also randomized across participants.

#### Design and procedure

Participants were randomly assigned to either a group that trained with words or a group that trained with pseudowords. In each group, participants were randomly assigned to one of the three different audiovisual training conditions: lipread speech, text, or a still face. In total, participants were thus assigned to six different groups in a 2 (Lexical status: words, pseudowords) * 3 (Audiovisual training: lipread, text, still face) between-subjects design. There were 21 participants in each group, except for the text group that received pseudoword training that contained 20 participants, as mentioned before.

Participants were seated in front of a 20-inch widescreen flat panel monitor (1680 × 1050 px resolution, 60 Hz refresh rate) in a sound attenuated booth and were instructed to attentively listen to the speech sounds while looking at the screen. Sounds were delivered through headphones (Sennheiser HD 203) at ~65 dBA (measured at ear level). The experiment was run using the E-prime 2.0 software (available at https://pstnet.com), and total testing lasted ~25 minutes. On all trials (training and test), participants typed in what they perceived, and this response was stored on the hard drive. Trials were automatically scored as ‘correct’ when the typed input exactly matched the original item. All incorrect responses were manually checked by a native Dutch speaker (MB) to find other orthographically ‘correct’ responses that were missed in the automatic procedure *(*e.g ‘noot’ [nut] spelled as the phonologically identical word ‘nood’ [need]: in Dutch, the voiced /d/ is pronounced as unvoiced /t/ when in final position).

**Fig 2:**
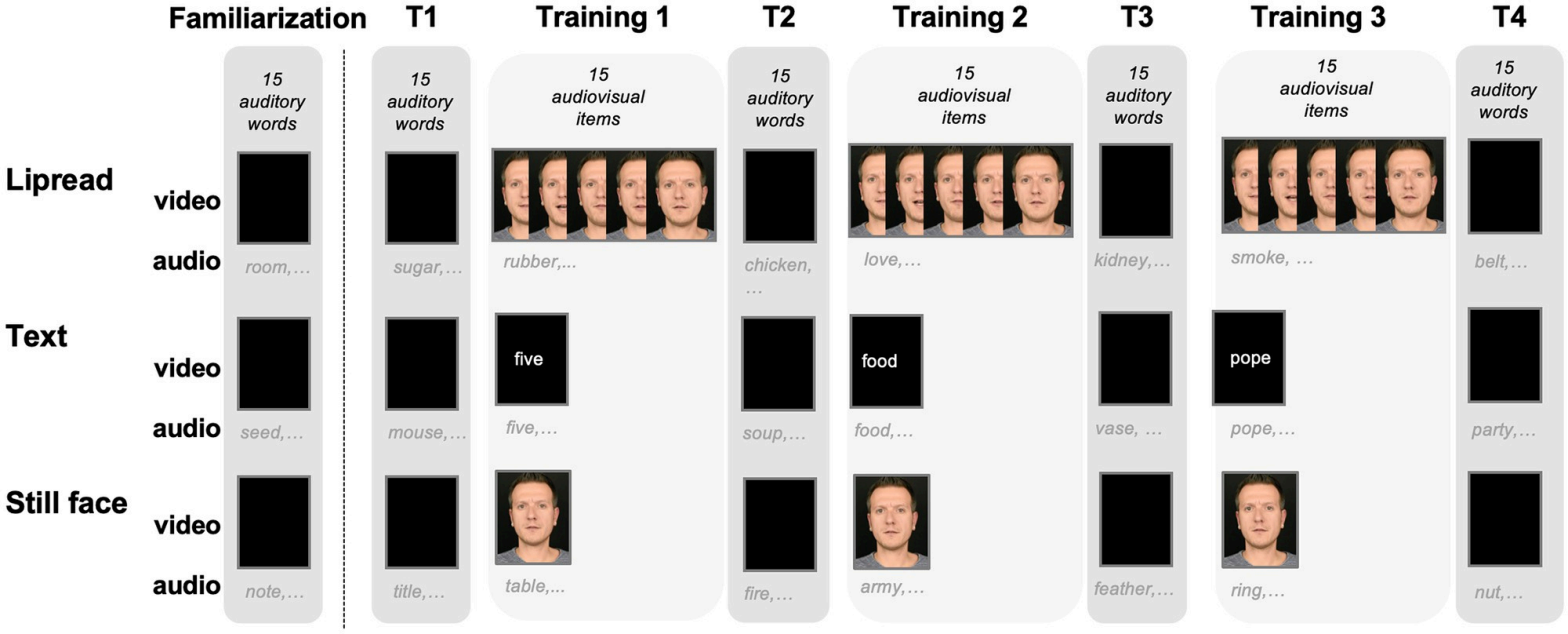
Experimental design. The familiarization phase and T1 consisted of 15 auditory words. Training blocks consisted of 15 audiovisual words or pseudowords, combined with either a dynamic face (i.e., lipread information), text, or a still face. Each training block was followed by an auditory-only test block (T2, T3 and T4) of 15 words. In total, there were three AV- training /Test blocks. After each training or test item, participants typed in what they had heard.

All participants first received a familiarization block (15 auditory-only trials) followed by a pretest (henceforth T1,15 auditory-only trials). Next, there were three AV training blocks (Training 1, Training 2, and Training 3; 15 items per block) interspersed with auditory-only test blocks named T2, T3, and T4 (15 trials per block, see Fig 2).

In total, 120 items were presented across 8 blocks of 15 unique items. Block order was counterbalanced across participants, except for the familiarization block, which was the same for all participants. In the pseudoword training groups, participants never received the two items from a particular word–pseudoword pair: if, for example, the pseudoword “pamer” (derived from the Dutch word “kamer” [room]) was presented during AV training, the word “kamer” was never used during the auditory test blocks. Auditory test words were randomly assigned to participants, and never repeated.

#### Analysis

All data were analyzed in R (version 4.10) using the lme4 package, version 1.1–27 [40]. Data were analyzed using generalized linear mixed effects models of the binomial family, which were fitted to the data by maximum likelihood estimation (Laplace Approximation) using the logit link function and the optimizer ‘bobyqa’. Significant main and interaction effects were further examined with post-hoc pairwise comparisons (two-tailed, Holm-Bonferroni corrected p values) on the model-predicted estimated means using the R package lsmeans (version 2.30–0).

### Results

**Fig 3:**
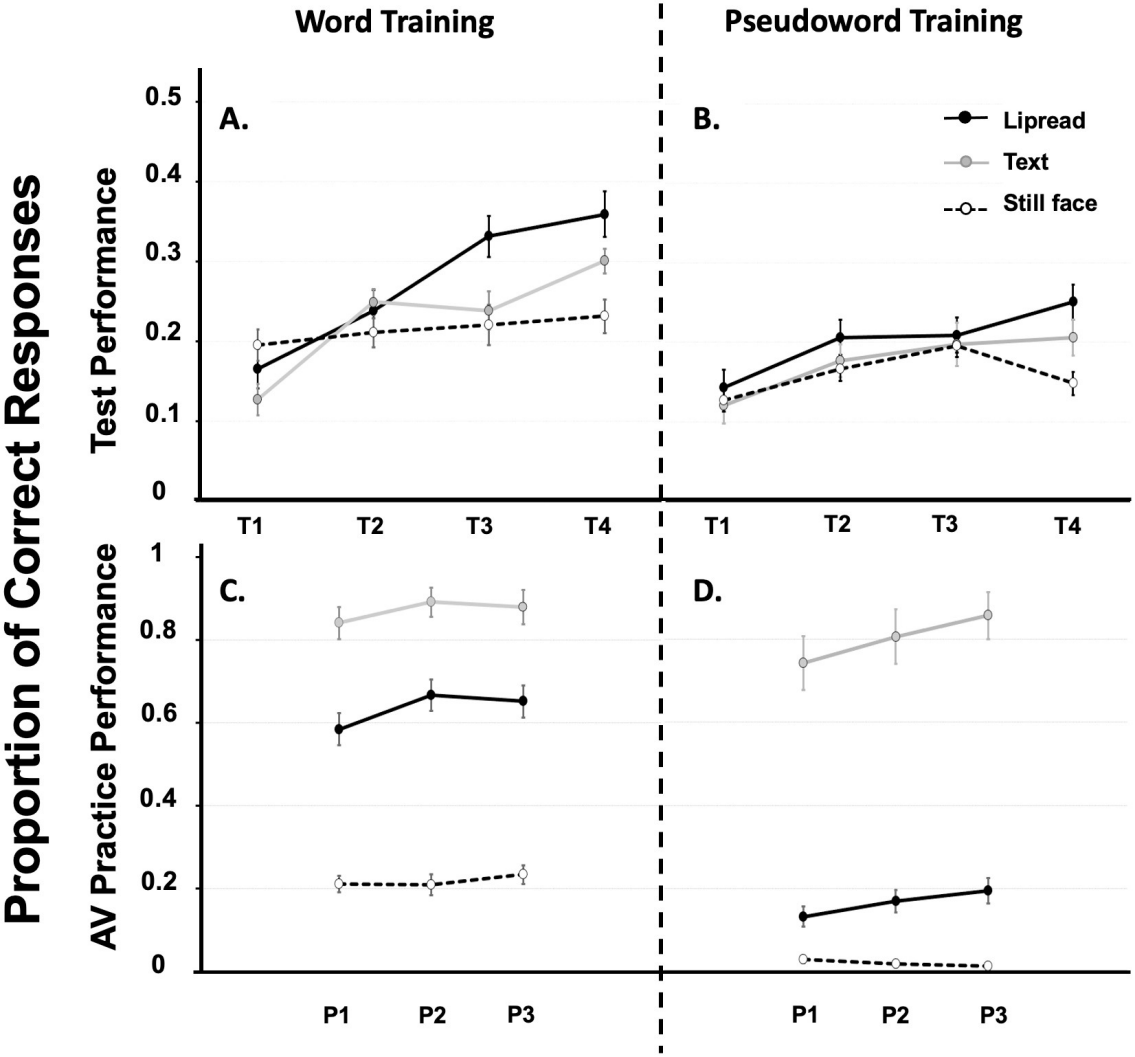
Proportion of correct responses during test and training blocks of Experiment 1. Grand average proportion of correct responses during test and training blocks for each type of training conditions (lipread speech, text, still face) and lexical status (words, pseudowords). The upper panels show accuracy of auditory-only word recognition during test blocks for participants trained with words (panel A) or pseudowords (panel B). The lower panels show accuracy during the audiovisual training blocks for words (panel C) or pseudowords (panel D). Error bars represent one standard error of the mean.

The grand average proportion of correct responses during test and training blocks for each type of training conditions (lipread speech, text, still face) and lexical status (words, pseudowords) are shown in Fig 3. Note that Accuracy at test is rather low, but as expected given that Pilling et al. [29] reported accuracy values at around 50% when participants identified keywords embedded in 8-channel auditory noise-vocoded sentences (as opposed to the 4-channel auditory noise-vocoded single items that we presented here) after AV training that included text or lipread speech.

#### Performance during auditory-only test blocks

Visual inspection of the data obtained from the auditory-only test blocks showed that overall performance was more accurate if participants were trained with words rather than pseudowords, a lexical boost of ~6 percentage points (i.e., ~3–4 items out of the total set of 60 A-only test items, see Fig 3, upper panels). Accuracy also improved across consecutive test blocks, and this effect was largest when participants were trained with lipread speech, intermediate when trained with text, and close to zero when trained with a still face.

To test these observations more formally, a generalized linear mixed effects logistic model was fitted to the data. The model included fixed effects for Training (lipread speech, text, still face), Lexical status (words, pseudowords) and Test block (1,2,3,4). We used the maximal random effect structure supported by the data, with by-subject and by-item random intercepts. Training type was dummy-coded such that training with a still face was set as the reference category for this factor. The factors Lexical status and Test block were recoded such that their values were centered around 0 (i.e. pseudowords and words were recoded into -1 and 1; T1, T2, T3 and T4 were recoded into -1.5, -0.5, 0.5 and 1.5). This ensured that all levels of Lexical status and Test block were considered in the fitted coefficients for the main effects and interactions including these factors. As a result, the fitted coefficient for Lexical status could be interpreted as the difference in correct responses (in log- odds) between training with words vs. pseudowords. Similarly, the fitted coefficient for Test block reflected the main effect of this factor. The fitted model was: Correct ~ 1 + Training type × Test block × Lexical status + (1 | subject) + (1 | item). Fixed effect coefficient estimates are shown in Table 1.

**Table 1:** Fixed effect coefficient estimates for the generalized linear mixed effects model fitted on the auditory-only test data. Correct ~ 1 + Training type × Test block × Lexical status + (1 | subject) + (1 | item).

Fixed factor	Estimate	*SE*	*z*-value	*p*
(Intercept)	-2.50	0.23	-10.85	< .001***
Training type _lipread speech	0.46	0.14	3.56	< .001***
Training type _text	0.12	0.14	0.85	.40
Test block	0.11	0.06	1.98	.048*
Lexical status	0.32	0.10	3.26	.001**
Training type _lipread speech × Test block	0.34	0.08	4.30	< .001***
Training type _text × Test block	0.27	0.08	3.33	< .001***
Training type _lipread speech × Lexical status	0.04	0.14	0.30	.76
Training type _text × Lexical status	-0.00	0.14	-0.07	.95
Test block × Lexical status	0.00	0.06	0.06	.95
Training type _lipread speech × Test block × Lexical status	0.11	0.08	1.48	.14
Training type _text × Test block × Lexical status	0.10	0.08	1.24	.22

* *p* < .05;

** *p* < .01;

*** *p* < .001,

SE: standard error.

The model revealed a significant main effect for the intercept (*b* = −2.50, SE = 0.23, *p* < .001), indicating an overall bias towards an incorrect response when participants were trained with a still face—which fits the overall response distribution (see Fig 3, upper panels). There was a main effect of Lexical status (*b* = 0.32, SE = 0.10, *p* = .001), indicating that overall test performance was higher when listeners were trained with words rather than pseudowords. Overall test performance was significantly higher after training with lipread speech when compared to training with a still face (*b* = 0.46, SE = 0.14, *p* < .001), while there was no difference in overall performance between training with text and a still face (*b* = 0.12, SE = 0.14, *p* = .40). In addition, there was a main effect of Test block (*b* = 0.11, SE = 0.06, *p* = .048), indicating that, on average, listeners were more likely to correctly identify the speech sounds with each successive test block. However, the model also showed that, compared to training with a still face, this improvement over time was larger when participants were trained with lipread speech (*b* = 0.34, *SE* = 0.08, *p* < .001) and text (*b* = 0.27, *SE* = .08, *p* < .001). There were no other significant main or interaction effects (*p*s > .13).

Post-hoc pairwise comparisons on the model-predicted means revealed that the effect of Test block was only significant for training with lipread speech and text (*p*s < .001), but not in the still face condition (*p* = .29). Importantly, these effects were not due to performance differences between training types during the first test block (*p*s > .38). After the third and final training block (i.e. at T3 and T4), performance after training with lipread speech was significantly higher than after training with a still face (T3: *b* = 0.63, SE = 0.14, *p* < .001, T4: *b* = 0.97, SE = 0.18, *p* < .001) or text (T3: *b* = 0.38, SE = 0.14, *p* = .014, T4: *b* = .45, SE = 0.17, *p* = .01). After the final training block (at T4), recognition performance was also significantly higher after training with text compared to training with a still face (*b* = 0.52, SE = 0.18, *p* = .007).

#### Performance during audiovisual training blocks

Visual inspection of the data from the training blocks showed that overall performance was more accurate if participants were trained with words compared to pseudowords (see Fig 3, lower panels). Intelligibility of the speech sounds was highest when they were accompanied by text, intermediate when accompanied by lipread speech, and lowest when a still face was presented simultaneously.

These observations were confirmed by a generalized linear mixed effects logistic model similar to the one fitted on the auditory-only test data. The model included fixed effects for Training type (lipread speech, text, still face), Lexical status (words, pseudowords) and Training block (1,2,3). The maximal random effect structure supported by the data was used, with by-subject and by-item random intercepts. Training type was dummy-coded such that training with a still face was set as the reference category. The factors Lexical status and Test block were recoded such that their values were centered around 0. The fitted model was: Correct ~ 1 + Training type × Training block × Lexical status + (1 | subject) + (1 | item). Fixed effect coefficient estimates are shown in Table 2.

**Table 2:** Fixed effect coefficient estimates for the generalized linear mixed effects model fitted on the training data. Correct ~ 1 + Training type × Training block × Lexical status + (1 | subject) + (1 | item).

Fixed factor	Estimate	*SE*	*z*-value	*p*
(Intercept)	-3.66	0.29	-12.42	< .001***
Training type _lipread speech	2.84	0.35	8.04	< .001***
Training type _text	6.66	0.39	17.15	< .001***
Training block	-0.13	0.17	-0.74	.46
Lexical status	1.74	0.29	5.98	< .001***
Training type _lipread speech × Training block	0.36	0.19	1.92	0.055
Training type _text × Training block	0.63	0.20	3.14	0.002**
Training type _lipread speech × Lexical contrast	-0.00	0.35	-0.003	.99
Training type _text × Lexical status	-1.39	0.37	-3.75	< .001***
Training block × Lexical status	0.21	0.17	1.27	.21
Training type _lipread speech × Training block × Lexical status	-0.23	0.19	-1.23	.21
Training type _text × Training block × Lexical status	-0.42	0.20	-2.11	.035*

* *p* < .05;

** *p* < .01;

*** *p* < .001,

SE: standard error

The model revealed a significant main effect for the intercept (*b* = −3.66, *SE* = 0.29, *p* < .001), indicating an overall bias towards an incorrect response when participants were trained with a still face. There was a significant main effect of Lexical status, *b* = 1.74, *SE* = 0.29, *p* < .001, indicating that recognition of words was noticeably better than recognition of pseudowords. Overall performance was higher during training with text (*b* = 6.66, *SE* = 0.39, *p* < .001) and lipread speech (*b* = 2.84, *SE* = 0.35, *p* < .001) when compared to the training with a still face. There were no other main or interaction effects (*p*s > .21).

Post-hoc pairwise comparisons on the model-predicted means showed that performance significantly differed between all three types of training (*p*s < .001), such that overall recognition performance was highest during training with text, intermediate during training with lipread speech, and lowest during training with a still face. There was a significant two-way interaction between training with text and Training block (*b* = 0.63, SE = 0.20, *p* = .002, suggesting an increase in accuracy over time during training with text. However, there was also a three-way interaction between training with text, Training block and Lexical status (*b* = -1.38, SE = 0.37, *p* < .001). Post-hoc simple effects analysis showed that the effect of Training block was significant for training with text and pseudowords (*p* < .001), but not for training with text and words (*p* = .33).

### Discussion

Experiment 1 showed that auditory learning of noise-vocoded speech was larger if listeners were trained with words rather than pseudowords (a *lexical* boost). Secondly, auditory learning of noise-vocoded speech was largest if listeners were trained with lipread speech (a lipread boost), intermediate if trained with text, and almost absent if trained with a still face. Words with lipread speech, rather than words-with-text, were thus the best guides for auditory speech learning. This is intriguing if one considers that during training, words-with-lipread speech were more *difficult* to recognize than words-with-text. So, despite the fact that lipread speech was more difficult to decode than text, it was nevertheless the best guide. Perhaps, the perceived difficulty of the AV lipread training trials (when compared to the AV text condition) has provided listeners with a more enganging or motivating learning context, but since previous work had revealed no differences between text and lipread training contexts [29], this tentative explanation of the current results requires an in-depth follow-up investigation. Presumably though, there are differences in binding–or integration–of text versus lipread speech with the audio, and we will return to this issue in the General Discussion.

The results from Experiment 1 further indicate that the learning effect in the lipread condition has built-up somewhat quicker than in the text condition: averaged across words/pseudowords, performance in the lipread condition was higher than in the text and still face condition in test blocks 3 and 4 (after receiving 30 AV training traisl), whereas performance in the text condition was higher than in the still face condition in test block 4 only (after receiving 45 AV training trials). So lipread speech not only provided stronger learning effects than text, but these effects also started earlier.

Early and rapid improvements in performance–as observed here–are mostly driven by perceptual learning (as opposed to procedural learning, see [41]), and such effects may dissipate quite quickly (i.e., auditory training regimes that produce longer-lasting (rehabilitation) effects typically span a longer time-frame [42]). However, because it is actually not clear whether the short-term auditory learning effects we observed vanish in minutes, or possibly reflect much longer-term effects lasting for days, weeks, or even months, we re-invited the participants from Experiment 1 that were originally trained with words (because they had the largest training effects and because this is the most natural situation encountered in real life) to test their auditory-only word recognition after a ~45-days rest period.

## Experiment 2

Participants from Experiment 1 who were originally trained with words were asked to return to the lab ~45 days later. During this session, they received no training and were thus only tested with the auditory-only noise-vocoded words they had heard in Experiment 1.

### Materials and methods

#### Participants

Participants from Experiment 1 who were trained with words were asked to return to the lab ~45 days later. The compliance rate was ~56% (N = 35, Mean age = 19.32, 29 females). There were 11 participants previously trained with lipread speech, 13 participants were trained with text, and 11 participants were from the baseline still face condition.

#### Stimuli

Stimuli were the same as in Experiment 1, but the 120 training and test words were now presented as auditory-only test items. Participants completed eight blocks of 15 audio-only trials. A familiarization block was followed by seven auditory-only test blocks (T1 to T7). Block order was randomized across participants, except for the familiarization block, which was the same across participants.

#### Design and procedure

Design and procedure were the same as in Experiment 1, except here three training blocks turned into test blocks; Participants completed eight blocks of 15 audio-only trials. A familiarization block was followed by seven auditory-only test blocks (T1 to T7). Block order was randomized across participants, except for the familiarization block, which was the same across participants.

#### Analysis

The data of the follow-up experiment were further analyzed in R using a generalized linear mixed effects logistic model similar to those fitted on the auditory-only test data and training data of experiment 1. We also compared the last test block in Experiment 1 (now referred to as T4E1, for test block 4, Experiment 1) with the first test block in Experiment 2 (T1) to assess retention of the learning effect between the two experiments. Significant main and interaction effects were further examined with post-hoc pairwise comparisons (two-tailed, Holm-Bonferroni corrected p values) on the model-predicted estimated means.

### Results

**Fig 4:**
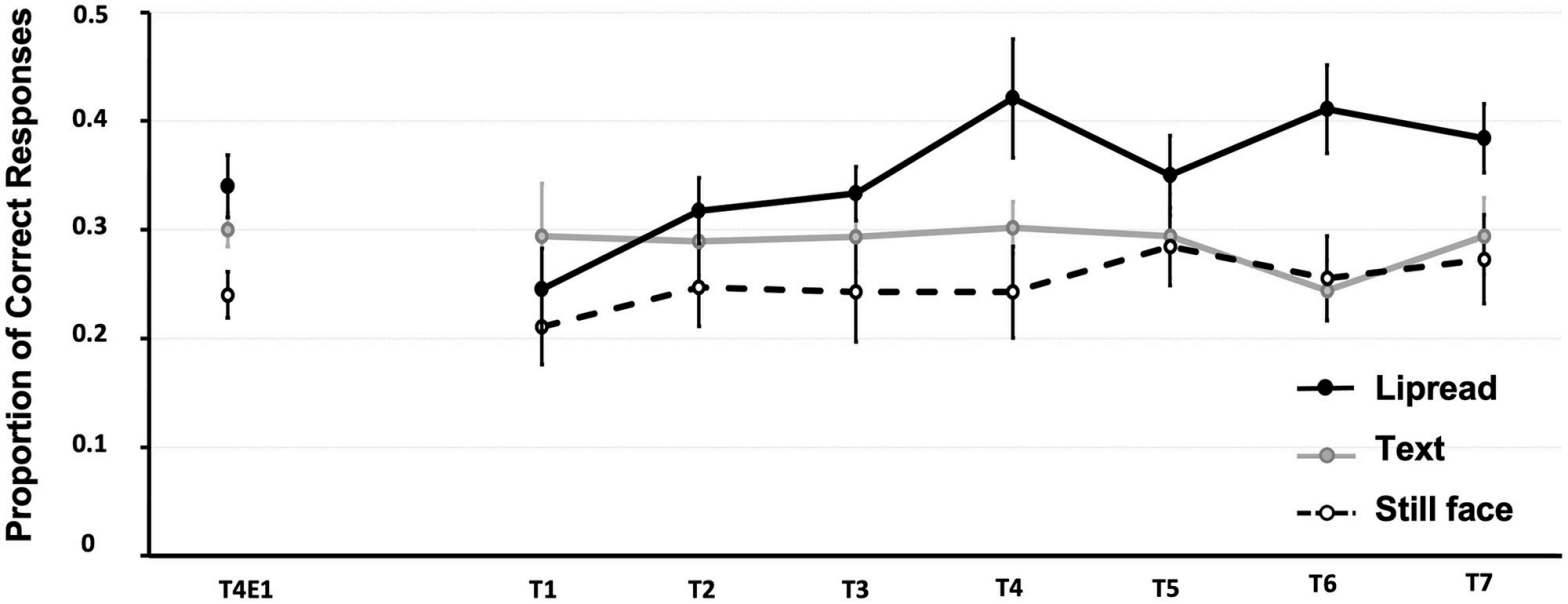
Proportion of correct responses in Experiment 2. Grand average proportion of correct responses during the follow-up auditory-only test blocks for each type of training conditions (lipread speech, text, still face). Data from the last test block from Experiment 1 (T4E1) is also included. Error bars represent one standard error of the mean.

The grand average proportions of correct responses during follow-up test blocks for each type of training conditions (lipread speech, text, still face) are shown in Fig 4.

Visual inspection of the data showed that overall performance during the follow-up test was more accurate if participants were previously trained with lipread speech rather than text or a still face. Accuracy also improved across consecutive test blocks for participants trained with lipread speech, while performance for those previously trained with text or a still face remained more constant across the test blocks.

A generalized linear mixed effects logistic model was fitted to the data to test these observations more formally. The model included fixed effects for Training type (lipread speech, text, still face) and Test block (1,2,3,4,5,6,7), with by-subject and by-item random intercepts. Training type was dummy-coded such that training with a still face was set as the reference category. The factor Test block was recoded such that all values were centered around 0. The fitted model was: Correct ~ 1 + Training × Test block + (1 | subject) + (1 | item). Fixed effect coefficient estimates are shown in Table 3.

**Table 3:** Fixed effect coefficient estimates for the generalized linear mixed effects model fitted on the auditory-only follow-up test data. Correct ~ 1 + Training type× Test block + (1 | subject) + (1 | item).

Fixed factor	Estimate	*SE*	*z*-value	*p*
(Intercept)	-1.89	0.29	-6.51	< .001***
Training type _lipread speech	0.89	0.29	3.05	0.002**
Training type _text	0.35	0.28	1.25	0.21
Test block	0.11	0.09	1.30	0.19
Training type _lipread speech × Test block	0.25	0.12	2.11	0.035*
Training type _text × Test block	-0.16	0.12	-1.39	0.16

* *p* < .05;

** *p* < .01;

*** *p* < .001,

SE: standard error

The model revealed a significant main effect for the intercept (*b* = −1.89, *SE* = 0.29, *p* < .001), indicating an overall bias towards an incorrect response when participants were previously trained with a still face. More importantly, overall performance during the follow-up test was significantly higher for participants who were previously trained lipread speech (*b* = 0.89, SE = 0.29, *p* = .002) compared to those previously trained with a still face. There was no difference in overall performance during follow-up between training with text and a still face (*b* = 0.35, SE = 0.28, *p* = .21). In addition, there was a significant two-way interaction between training with lipread speech and Training block (*b* = 0.25, SE = 0.12, *p* = .035, indicating an increase in accuracy with each consecutive test block for participants previously trained with lipread speech. There were no other main or interaction effects (*p*s > .16).

Interestingly, post-hoc pairwise comparisons showed that performance during the first two follow-up test blocks did not significantly differ between training types (*p*s > .13). However, participants previously trained with lipread speech showed a significant increase in performance over those who were trained with a still face and text, from the third until the final test block (*p*s < .04) and from the fifth until the final test block, respectively (*p*s < .02).

The model that included the last test block from experiment 1 (T4E1) and T1 from Experiment 2 revealed a main effect of Intercept, (*b* = −2.16, SE = 0.37, *p* < .001), indicating an overall bias towards an incorrect response for participants who were trained with a still face. However, no significant main effect of Test block was observed, (*b* = -0.02, SE = 0.12, *p* = .89), indicating that the overall learning effect across conditions in Experiment 1 was retained during the period in between Experiments 1 and 2. However, there was no significant main effect of Training type, nor a significant Training type × Test block interaction, *p*s >.053, indicating that at the start of Experiment 2, performance of participants from the different groups (text, lipread information and still image) was statistically comparable (see Fig 4).

### Discussion

Experiment 2 demonstrated that lipread speech was also at longer-term the best guide to auditory learning: Listeners who were previously trained with lipread speech outperformed listeners who were previously trained with text or with a still face when tested ~45 days later. More specifically, participants in the lipread group outperformed those from the text group from the fifth test block onward (after 60 A-only test items) and outperformed those from the still face group from the third test block onward (after 45 A-only test items), whereas performance in the text group was statistically comparable to the still face group across all test blocks.

In Experiment 1, we thus established that lipread information provided a better visual teaching context than text (both in effect size and onset of the effect), and Experiment 2 showed that this lipread-induced advantage transferred to an auditory-only test administered ~45 days later.

## General discussion

Distorted speech can be difficult to understand, but under the right circumstances, listeners quickly adapt to this situation and thus become better listeners. These adaptive changes, here referred to as learning effects, are boosted if other information sources, like prior word knowledge, text, or lipread speech are available to disambiguate the acoustics. Here we examined the relative contribution of these extra information sources to auditory learning on noise-vocoded speech. Listeners were trained with auditorily-distorted words or pseudowords that were made clearer by combining them with either lipread speech or text that was presented simultaneously with these auditory items. After short audiovisual training sessions, listeners were tested with novel auditorily-only distorted words. Results showed that training with words boosted auditory learning relative to training with pseudowords (a lexical boost). This effect did not interact with whether the training context was lipread information, text, or a still face. This is in-line with previous work that showed that lipread information and lexical context may operate simultaneously during speech processing but seem to do so independently [43], presumably because lipread context affects the immediate percept, but does not directly affect linguistic encoding as lexical information does [44, but see 45, for comparable selective adaptation effects induced by lexical and lipread information].

In words and pseudowords alike, both text and lipread speech boosted auditory learning relative to a static control condition. Most remarkably, training with lipread speech was more effective than training with text (a lipread boost), despite the fact that distorted items with lipread speech were more difficult to decode during training than items with text. This lipread boost was also surprisingly long-lasting as it was still present when participants were tested more than a month later. Words combined with lipread speech, the natural situation in face-to-face conversation, thus proved to be the best guide for speech learning at short- and long-term.

To be clear, the test items in Experiment 2 were the same items that participants had previously received in Experiment 1 (either during test or training), so it is theoretically possible that lexical items were committed to memory during Experiment 1, which then may produce a transfer effect (retrieval from memory) to Experiment 2. In Experiment 1, training with text induced a higher overall proportion of correctly identified items than training with lipread speech (combined across training and test blocks), and in the AV training blocks, participants in the text condition essentially received more accurate (orthographic) feedback about the identity of the item than participants in the lipread and still face conditions. Therefore, it stands to reason that participants in the text condition should have an encoding advantage in Experiment 1, and potential retrieval from memory would thus have been higher in the text condition than in the lipread and still face conditions. This was not the case, as in Experiment 2, participants who were previously trained with lipread speech outperformed those who were previously trained with text (the text group did not perfome differently from participants who were previously trained with a still face). Therefore, a general memory recall mechanism provides a rather unsatisfying explanation for performance in Experiment 2.

It is also unlikely that the learning effect itself took place on a lexical level, because learning effects in NVS seem to be constituted on a pre-lexical rather than lexical level [28]. This then begs the question why learning on a pre-lexical level was stronger when the visual training context contained lipread information rather than text. One possibility is that during training, participants were paying more attention to the sound when it was combined with lipread information rather than text, because in the latter condition, participants could produce the correct answers by simply typing what they had seen. Future work should therefore examine if paying attention to sound indeed contributes to auditory speech learning. For example, including catch trials in which sound and visual information are incongruent, could increase participants‘ attention during the task.

Our findings are in line with many other studies that reported that auditory learnig can be boosted if extra information is provided that disambiguates the acoustics of distorted words. This extra information may come from the internally generated lexical guesses that are likely generated if the acoustics are not too distorted [14], or from external feedback-like text or lipread speech that provide complementary information about the intended acoustic message, for review see [6]. There is also a general consensus in the literature that, whatever is learned during training, there is transfer of auditory learning effects to other words not encountered before [14, 46, 47]. In fact, in the present study (i.e., in Experiment 1), there was no overlap between training and test items. This suggests that even though lexical knowledge boosts auditory learning, the adaptive changes themselves indeed occur in the mapping of the distorted sounds to pre-lexical representations as noted before, see also [28].

The results also showed that during training, items were much easier to recognize when accompanied by text than lipread speech. This is hardly surprising, because reading–for an adult–is after all very easy, while lipreading can be hard. In essence, then, lipread speech contained *less* phonetically relevant information than text. What is surprising, then, is that lipread speech nevertheless induced *larger* auditory learning effects than text. From a computational point of view, this seems at odds with models of supervised learning where the magnitude of learning depends on both the quality of the input relative to the size and reliability of the disambiguating context [48]. In these models, adaptation/recalibration is driven by the interaction between these two factors to generate an error signal that drives learning. When the teacher signal itself becomes more ambiguous, one would expect adaptation/recalibration to become smaller. If one assumes that–in a transparent language–ortographic information provides an unambiguous context that perfectly maps onto phonetic representations, whereas such mapping driven by lipread information is more ambiguous, one may expect lip-read induced adaptation/recalibration to be smaller, and not larger, than text-induced effects.

Our results are in line with studies on phonetic recalibration where it has been found that the size of the recalibration effect is larger when induced by lipread speech than text [15]. Lipread speech is often also more powerful than lexical information when driving phonetic recalibration [16, 20], and these findings thus all raise the question why lipread speech, compared to text or lexical information, is so effective at driving auditory learning and recalibration. One potential explanation is provided by an electrophysiological (EEG) study conducted Stekelenburg et al. [49]. In that study, the audiovisual Mismatch Negativity (MMN: a component of the event‐related potential reflecting pre-attentive auditory change detection) was investigated. More specifically, deviant text or deviant visual speech was used to induce an illusory change in a sequence of ambiguous standard sounds halfway between /aba/ and /ada/. At the behavioral level, it was found that lipread speech was more than 4 times as effective than text in biasing identification of the ambiguous sound toward the visual stimulus (a shift of 93% for lipread speech versus 20% for text). But most importantly, only deviant visual speech induced an MMN, but not deviant text (which induced a late P3‐like positive potential), suggesting that lipread speech, but not text, can change sound processing at a pre-attentive level.

One factor that may explain this, is that the dynamics of the moving lips and the unfolding sound are tightly coupled in time, which is not the case for text. On a behavioral level, it has been demonstrated that an orthographic prime (followed by an auditory target sentence) lowers the detection threshold of speech masked in noise, but not to the same degree as an AV stimulus in which lipread information is presented simultaneously with the audio, presumably because the temporal coherence between unfolding lipread information and speech facilitates speech detection [50]. Electrophysiological investigations have revealed that integration of temporal (and spatial) information in an AV speech signal seems to occur prior to integration on a phonetic level [51–56]. So even though we had suggested that phonetic binding of the noise-vocoded input with a visual context might be less prone to errors when the visual context comprises text rather than lip-read information, it is possible that integration of lower-level temporal stimulus features–that occurs before phonetic integration–is a prerequisite for optimal phonetic binding. If so, the relatively weak temporal relationship between an orthographic item presented on a screen and an unfolding noise-vocoded sound may have resulted in relatively weak phonetic binding, which–in turn–would lead to weaker learning effects for text when compared to the lipread context. However, a direct comparison between both types of visual information with a technique that can track the different stages of integration (such as EEG) is needed to develop this line of thought more definitively.

Hickok et al. [57] proposed the dual-stream processing model of speech. According to the model, areas of the brain along a ventral pathway, including medial temporal gyrus (MTG) and inferior temporal sulcus (ITS), are geared towards connecting phonological and lexical representations, while regions along a dorsal pathway, including parietal-temporal, (pre)motor, and inferior frontal regions, are geared towards connecting phonological with sensorimotor and articulatory representations. Adank et al. [58] explored how listeners adjust to recordings of unclear sentences, and found activation patterns consistent with the Hickok & Poeppel model [57]. Holdgraf et al. [59] also found evidence for acoustic updating, using spectro-temporal receptive field mapping on ECoG recordings of the auditory cortex. Responses of cortical populations were observed to have increased sensitivity to speech-like spectro-temporal features of degraded speech, after exposure to intact speech. This sensitivity could reflect how listeners encode rudimentary acoustic features that allow the listener to interpret less intelligible speech, and how listeners adapt to distorted speech. In a functional Magnetic Resonance Imaging (fMRI) study on lipread recalibration, Kilian-Hutten et al. [60] noted functional connectivity between occipital regions and left auditory cortex during lipread induced recalibration. Furthermore, it appeared that recalibration induced by lipread speech induced changes in speech perception that were measurable as subtle changes in auditory cortical activity [13, 60]. In a recent study on lipread versus lexical phonetic recalibration [23], behavioral results showed that lipread speech was more than twice as effective than lexical information driving recalibration. At the neuronal level, it appeared that lipread speech and lexical information both induced largely similar brain response patterns. However, lipread recalibration also involved additional visual cortex, suggesting that previously acquired visual information on lip movements was retrieved and deployed to disambiguate auditory perception. Thus, following lipread as well as text-based recalibration, it is possible to retrieve participant’s perceptual interpretation of the ambiguous speech sounds from posterior auditory cortical activity patterns, indicating that both types of inducer stimuli can serve a disambiguating role in phonetic adjustments, but lipread recalibration also involves additional visual cortex.

To conclude, we found that the optimal signal to drive auditory learning of noise vocoded speech were words combined with lipread speech. Fortunately, this approximates real-life situations in which we see and hear a speaker talking. It remains to be examined if these results hold if longer segments than isolated words—like phrases or sentences—are used. Possibly, these findings may provide a platform to (further) develop treatments for people with hearing and learning problems, especially for young children with cochlear implants (CIs) who have not yet learned to read, or for children who have difficulties in reading [32]. As noted, though, training regimes designed for rehabilitation purposes typically include longer training sessions and span a longer time-window than the approach we used in current inverstigation [42]. However, Hawkey et al. [41] argued that rapid and early learning–which is exactly what we observed here–is actually critical for training programs designed to counter auditory deficits. To reconcile the above, future work should therefore first determine the boundaries of the rapid learning effects we observed in more detail (in terms of ceiling effects and robustness over time), and assess the degree to which these effects may generalize to other types of stimuli (that yield higher overall performance or include different types of signal degradation) or populations, such as CI-users.

## Supporting information

S1 Table Stimuli list. Words and pseudowords used during test (words) and training (words and pseudowords).
(DOCX)
