# Supplementary material for: Want to quickly adapt to distorted speech and become a better listener? Read lips, not text
Source: PLoS One. 2022 Dec 29;17(12):e0278986. doi: 10.1371/journal.pone.0278986 (PMC9799298; doi:10.1371/journal.pone.0278986)
Supplement: S1 Table — Words and pseudowords used during test (words) and training (words and pseudowords). (DOCX) [file pone.0278986.s001.docx]

Table S1.

Item overview (words and derived pseudowords), their English translation (words only) and their frequency of occurrence (words only).

| **Dutch word [*English translation]*** | **Frequency per million** | **Derived pseudoword** |
| --- | --- | --- |
| kamer [*room*] | 275.24 | pamer |
| neef [*cousin*] | 48.48 | feen |
| zaad [*seed*] | 6.33 | taaf |
| val [*trap*] | 115.46 | sal |
| zebra [*zebra*] | 3.06 | breza |
| toeval [*coincidence*] | 29.75 | loevat |
| boef [*crook*] | 6.20 | koes |
| fut [*pep*] | 0.34 | kuf |
| vak [*section*] | 22.02 | sak |
| boog [*bow*] | 8.55 | goop |
| bom [*bomb*] | 51.32 | nom |
| riem [*lash*] | 14.16 | liem |
| merel [*remel*] | 0.14 | blackbird |
| noot [*nut*] | 3.82 | koon |
| pool [*pole*] | 3.54 | poor |
| veer [*feather*] | 3.43 | reef |
| fik [fire] | 6.49 | zik |
| motor [*engine*] | 42.63 | rotom |
| gif [*poison*] | 13.56 | fig |
| vuur [*fire*] | 100.57 | ruuf |
| burger [*civilian*] | 19.19 | gurber |
| vijf [*five*] | 281.57 | vijp |
| taal [*language*] | 36.29 | laak |
| zomer [*summer*] | 42.90 | zorem |
| bar [*bar*] | 53.83 | tar |
| leraar [*teacher*] | 29.66 | relaar |
| les [*lesson*] | 66.43 | res |
| shampoo [*shampoo*] | 3.06 | zanto |
| sap [*juice*] | 7.09 | vas |
| gaas [*gauze*] | 0.82 | saas |
| sein [*signal*] | 3.11 | neis |
| tafel [*table*] | 83.40 | talef |
| folder [*flyer*] | 1.81 | lofder |
| mok [*mug*] | 1.12 | mog |
| raad [*councel*] | 83.01 | laap |
| pas [*step*] | 325.80 | fap |
| piek [*peak*] | 4.94 | biek |
| saus [*sauce*] | 9.56 | vaus |
| kop [*kead*] | 229.23 | pok |
| voeg [*joint*] | 5.03 | goef |
| fuif [*party*] | 2.61 | zuif |
| put [*well*] | 11.69 | tup |
| zeef [*sieve*] | 0.57 | fees |
| baby [*baby*] | 151.80 | byba |
| nek [*neck*] | 57.72 | kem |
| kater [*tomcat*] | 7.29 | batel |
| cheque [*cheque*] | 25.15 | chep |
| bon [*receipt*] | 10.34 | pon |
| muis [*mouse*] | 11.14 | suin |
| titel [*title*] | 18.23 | litet |
| satan [*satan*] | 8.80 | sanat |
| leger [*army*] | 107.98 | releg |
| fan [*fan*] | 19.21 | nef |
| reep [*strip*] | 2.65 | leep |
| zes [*six*] | 199.84 | zef |
| keizer [*emperor*] | 27.33 | beizer |
| pijp [*tube*] | 13.81 | bijp |
| taak [*task*] | 42.63 | kaat |
| zeep [*soap*] | 14.22 | zeek |
| rel [*riot*] | 2.70 | ler |
| boom [*tree*] | 52.25 | moop |
| feit [*fact*] | 43.04 | teif |
| negen [*nine*] | 64.28 | neneg |
| tak [*branch*] | 8.48 | bap |
| nagel [*nail*] | 4.05 | ganel |
| zeil [*sail*] | 6.91 | leis |
| poging [*attempt*] | 22.57 | goping |
| rug [*back*] | 80.79 | gur |
| fabel [*fabel*] | 0.53 | falep |
| muur [*wall*] | 66.89 | ruum |
| tempel [*temple*] | 14.66 | lempet |
| parel [*pearl*] | 3.02 | barel |
| zuivel [*dairy*] | 0.64 | zuilef |
| lach [*laugh*] | 46.72 | gar |
| liefde [*love*] | 208.90 | field |
| zet [*move*] | 315.55 | pes |
| gang [*corridor*] | 110.80 | dang |
| mug [*mosquito*] | 2.26 | nug |
| soep [*soup*] | 17.84 | voep |
| toon [*tone*] | 34.67 | nook |
| rubber [*rubber*] | 4.37 | lubbel |
| pit [*pit*] | 5.53 | bik |
| ring [*ring*] | 52.34 | rink |
| nier [*kidney*] | 3.91 | neil |
| beek [*stream*] | 3.43 | peek |
| nis [*niche*] | 0.96 | sin |
| moed [*courage*] | 41.05 | noet |
| regen [*rain*] | 26.48 | gener |
| bezem [*broom*] | 3.80 | zebem |
| naad [*seam*] | 1.78 | naag |
| koffer [*suicase*] | 33.87 | roffek |
| zoen [*kiss*] | 9.99 | voen |
| zuurkool [*sauerkraut*] | 0.80 | fuurbool |
| chic [*chic*] | 31.83 | bif |
| gevel [*façade*] | 0.41 | leveg |
| kom [*bowl*] | 2612.54 | pom |
| faam [*fame*] | 1.21 | maaf |
| zak [*bag*] | 96.87 | vap |
| ruzie [*quarrel*] | 64.19 | zurie |
| maag [*stomach*] | 23.55 | gaam |
| gil (*scream*) | 9.99 | gir |
| vogel (*bird*) | 32.27 | voleg |
| vat [*barrel*] | 19.05 | taf |
| fout [*error*] | 165.27 | touf |
| vaas [*vase*] | 4.57 | saaf |
| gordel [*seatbelt*] | 9.49 | rogdel |
| Voedsel [*food*] | 4.37 | doefsel |
| kip [*chicken*] | 37.89 | kif |
| kuif [*crest*] | 0.23 | tuif |
| set [*set*] | 14.25 | tef |
| boel [*lot*] | 52.37 | loet |
| paus [*pope*] | 12.62 | saup |
| bende [*gang*] | 32.24 | pende |
| rook [*smoke*] | 46.63 | toor |
| suiker [*sugar*] | 21.82 | fuiger |
| nummer [*number*] | 202.70 | nurrem |
| sinas [*soda*] | 0.57 | nisas |
| zoemer [*buzzer*] | 0.75 | zorem |
| chic [*chic*] | 4.37 | bif |
| sik [*goatee*] | 0.46 | gik |

*Note*. Only words were presented during the auditory test phases (Experiments 1 and 2), whereas words and pseudowords (a between-subjects factor) were used during AV training in Experiment 1.
